# Searching ChIP-seq genomic islands for combinatorial regulatory codes in mouse embryonic stem cells

**DOI:** 10.1186/1471-2164-12-515

**Published:** 2011-10-20

**Authors:** Gong Chen, Qing Zhou

**Affiliations:** 1 Department of Statistics, University of California, Los Angeles Los Angeles, USA

## Abstract

**Background:**

To facilitate deciphering underlying transcriptional regulatory circuits in mouse embryonic stem (ES) cells, recent ChIP-seq data provided genome-wide binding locations of several key transcription factors (TFs); meanwhile, existing efforts profiled gene expression in ES cells and in their early differentiated state. It has been shown that the gene expression profiles are correlated with the binding of these TFs. However, it remains unclear whether other TFs, referred to as cofactors, participate the gene regulation by collaborating with the ChIP-seq TFs.

**Results:**

Based on our analyses of the ES gene expression profiles and binding sites of potential cofactors in vicinity of the ChIP-seq TF binding locations, we identified a list of co-binding features that show significantly different characteristics between different gene expression patterns (activated or repressed gene expression in ES cells) at a false discovery rate of 10%. Gene classification with a subset of the identified features achieved up to 20% improvement over classification only based on the ChIP-seq TFs. More than 1/3 of reasoned regulatory roles of cofactor candidates involved in these features are supported by existing literatures. Finally, the predicted target genes of the majority candidates present expected expression change in another independent data set, which serves as a supplementary validation of these candidates.

**Conclusions:**

Our results revealed a list of combinatorial genomic features that are significantly associated with gene expression in ES cells, suggesting potential cofactors of the ChIP-seq TFs for gene regulation.

## Background

A set of core transcription factors (TFs) have been reported to regulate the self-renewal and pluripotency of mouse embryonic stem (ES) cells. Oct4 has long been regarded as one of the master regulators in ES cells. Oct4-deficient embryos fail to produce pluripotent inner cell mass [1]. Furthermore, while repression of Oct4 allows trophectoderm development, a less-than-twofold increase in Oct4 expression drives differentiation into primitive endoderm and mesoderm [2]. Together with Oct4, Sox2 explains the first three lineages present in preimplantation development; both factors are essential to epiblast formation, and in their absence trophec-toderm is formed [3]. Nanog, another master factor, can bypass leukemia inhibitory factor (LIF)/STAT3 to maintain ES cell self-renewal [4,5], and Nanog-deficient ES cells lose pluripotency and differentiate into extraembryonic endoderm lineage [5].

In addition to the three master TFs, implications of the regulatory roles of a few other TFs in mouse ES cells have been obtained via experimental efforts. LIF signal pathway can sustain self-renewal of the cells by activating STAT3 [6]; BMPs collaborate with LIF for the maintenance of self-renewal via triggering the phosphorylation of Smad1 to induce Id genes [7]. Myc and Klf4 are two of the four factors that can reprogram somatic cells to pluripotent cells [8]. Esrrb is required for efficient self-renewal of ES cells *in vitro*; it is required to block differentiation into mesoderm, ectoderm and neural crest cells [9]. Depletion of Zfx impairs self-renewal of ES cells while over-expression of the factor can facilitate the self-renewal [10].

**Figure 1:**
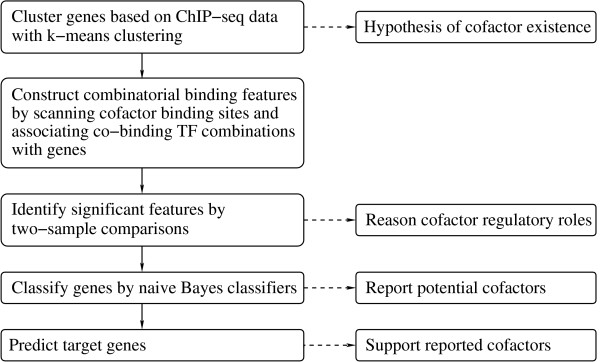
**The diagram of the analysis procedure**. The main steps are listed on the left, and their corresponding results/purposes are indicated on the right.

To reconstruct the regulatory network in mouse ES cells, genome-wide binding data of these important TFs have been generated by ChIP-seq/chip experiments [11-13]. In particular, Chen et al. [11] made available ChIP-seq data of the following 12 TFs in mouse ES cells: Oct4, Sox2, Nanog, STAT3, Smad1, Myc, Klf4, Zfx, Esrrb, Mycn, Tcfcp2l1, and E2f1. We refer to them as *main factors*. The authors showed that the binding of these factors is correlated with retinoic-acid-induced gene expression profiles [9]. Furthermore, Ouyang et al. [14] built statistical models upon the ChIP-seq data, which can explain substantial variation in gene expression in mouse ES cells [15,16]. However, the following problem remains to be investigated: Whether other TFs that bind together (or co-bind) with main factors, referred to as *cofactors*, can further explain expression patterns? In this study, with the ChIP-seq data in [11] and the expression data in [16], we attempt to answer this question by exploring association between the gene expression and binding sites of potential cofactors on genomic islands co-occupied by groups of main factors. We intend to evaluate whether the association is different between different expression patterns, and according to the evaluation, we recommend a small set of cofactors for future follow-up experimental validation. To this end, we perform the following analyses (Figure 1) and report their results in this article.

1. Clustering analysis of the ChIP-seq data and the gene expression profiles revealed that genes sharing similar binding patterns of the main factors may still have distinctive expression patterns, leading to our hypothesis of existence of cofactors. The *k*-means clustering was employed in this step.

2. We then constructed features characterizing co-binding effects of main factors and a cofactor candidate on their potential target genes. In order to integrate genomic information of cofactor candidates with the main factor binding data, we scanned genome regions defined by the ChIP-seq coordinates with a set of known TF motifs to identify co-localization of binding sites of main factors and a cofactor candidate in the genome; next, we computed a feature score for a gene and a feature--a combination of (co-localized) main factors and a cofactor candidate--such a score intends to quantify association strength between a gene and a co-binding combination, giving a numerical summary of the genomic sequence information and the ChIP-seq data for the gene.

3. Through hypothesis tests, we identified features which have significantly different distributions between ES-up genes and ES-down genes representing genes up- and down-regulated in ES cells, respectively (see Results for their definitions). By checking expression profiles of a cofactor candidate involved in a feature, we reasoned its regulatory role based on test results. Two-sample proportion test and the Wilcoxon rank-sum test were used, with the false discovery rate (FDR) controlled by the Benjamini-Hochberg procedure [17].

4. Regarding ES-up and ES-down genes as two classes of genes and significant features identified in the last step as predictors, we adopted gene classification with naïve Bayes classifiers to further choose a small subset of features, which suggest cofactor candidates through involved TF combinations.

5. Finally, we predicted target genes of TFs involved in selected features by perturbing learned classifiers and examining change in classification. We reported consistent evidence supporting our prediction from another independent data set. The purpose of this practice is to provide supplementary validation of cofactor candidates revealed by selected features via the target prediction.

For brevity, we refer to cofactor candidates as cofactors.

## Results

### Hypothesis of existence of cofactors

We first define gene sets of our interest and introduce some existing findings about the main factors based on the ChIP-seq data, and then present their interplay results to motivate our hypothesis of existence of cofactors and further analyses.

#### ES-up and ES-down genes

Zhou et al. [16] produced gene expression profiles at ES-cell stage and early differentiated stage, including 3 profiles of undifferentiated ES cells (with high Oct4 expression), 5 profiles of 2-, 4-, 8-day embryoid bodies with high Oct4 expression, and 8 profiles of 2-, 4-, 8-, 15-day embryoid bodies with low Oct4 expression. Regarding high Oct4 expression as a marker for ES cells, we refer to the 8 profiles with high Oct4 expression as samples at *ES stage *and the remaining profiles as samples at *DF (differentiation) stage*. Two gene sets have been identified: The genes whose expression is higher at ES stage than at DF stage with a fold change > 2 and *P*-value < 0.05 in a two-sample comparison, and the genes that have significantly lower expression at ES stage comparing with expression at DF stage under the same cutoffs. We refer to the first set of genes as *ES-up genes *and the second set of genes as *ES-down genes*. We call these two sets of genes collectively *ES genes*.

#### The Myc group and the Oct4 group of the main factors

In the ChIP-seq data [11], a pair of start and end coordinates specifies a peak location or a binding site of a TF in coarse resolutions, and the number of reads (after normalization) associated with a peak indicates the intensity of binding signals on the site. The authors showed that Oct4, Sox2, Nanog, Smad1, and STAT3 have different binding patterns from Myc and Mycn. Ouyang et al. [14] reported a similar phenomenon. Specifically, they first defined an association score to measure association strength between a gene and a main factor (see Methods). Intuitively, the closer the binding sites (peaks) of a main factor to the transcription start site (TSS) of the gene, and the stronger the ChIP-seq signal intensities (the number of reads) on the sites, the higher the association score between the gene and the main factor. Through principal components analysis of the association scores of all the main factors, they identified two groups of TFs: *The Myc group *consists of E2f1, Zfx, Mycn, and Myc; *the Oct4 group *has Oct4, Sox2, Nanog, Smad1, STAT3, Tcfcp2l1, and Esrrb. While the Myc group is mainly associated with up-regulated genes in ES cells, the Oct4 group may either activate or repress genes at ES stage according to their regression analysis of the association scores and expression data.

#### Gene clusters based on association scores

**Figure 2:**
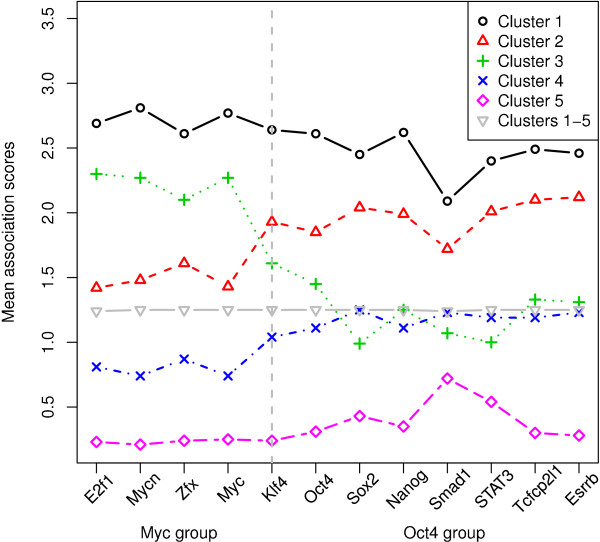
**Mean association scores of gene clusters**. Each data point in the figure indicates the mean of the association scores of a cluster of genes for a TF. The vertical broken line separates the Myc group and the Oct4 group. "Clusters 1-5" indicates the grand mean scores of all the genes for a TF.

Having learned the differences between these two TF groups, we intended to examine whether genes can be reasonably partitioned according to the association scores. This examination was to check whether a similar binding pattern shared by a set of genes would lead to similar expression of the genes. We applied the *k*-means clustering to genes based on their association scores with the 12 main factors (reported in [14]), and chose *k *= 5 as we may expect four clusters of genes associated with the binding of none/either/both of the two groups, leaving an additional cluster accommodating any unexpected binding pattern. Figure 2 shows the mean association scores for the five clusters and the grand mean association scores over all the genes (note that the grand means of different TFs are all around 1.25 because of quantile normalization in [14]). We observed distinguished binding patterns among these five clusters. By comparing the mean association scores with the grand means, we see that cluster 1 includes genes highly associated with all the TFs; in contrast, cluster 5 consists of genes having weak association with all of them. Cluster 2 has genes more intensively associated with the Oct4 group than the Myc group; in cluster 3, conversely, the Myc group shows stronger association than the Oct4 group; similar to cluster 2, cluster 4 contains genes for which the Oct4 group owns higher association scores than the Myc group, although the association is relatively moderate comparing with the association for cluster 2. For future reference, we name the five clusters according to the observed binding patterns: *The uniformly-high cluster *for cluster 1, *the uniformly-low cluster *for cluster 5, *the Oct4 cluster *for cluster 2, *the Myc cluster *for cluster 3, and *the Oct4-moderate cluster *for cluster 4. We excluded the uniformly-low cluster from further investigation because we were interested in finding cofactors working with the main factors in ES cells and all the main factors are weakly associated with the genes in that cluster.

**Figure 3:**
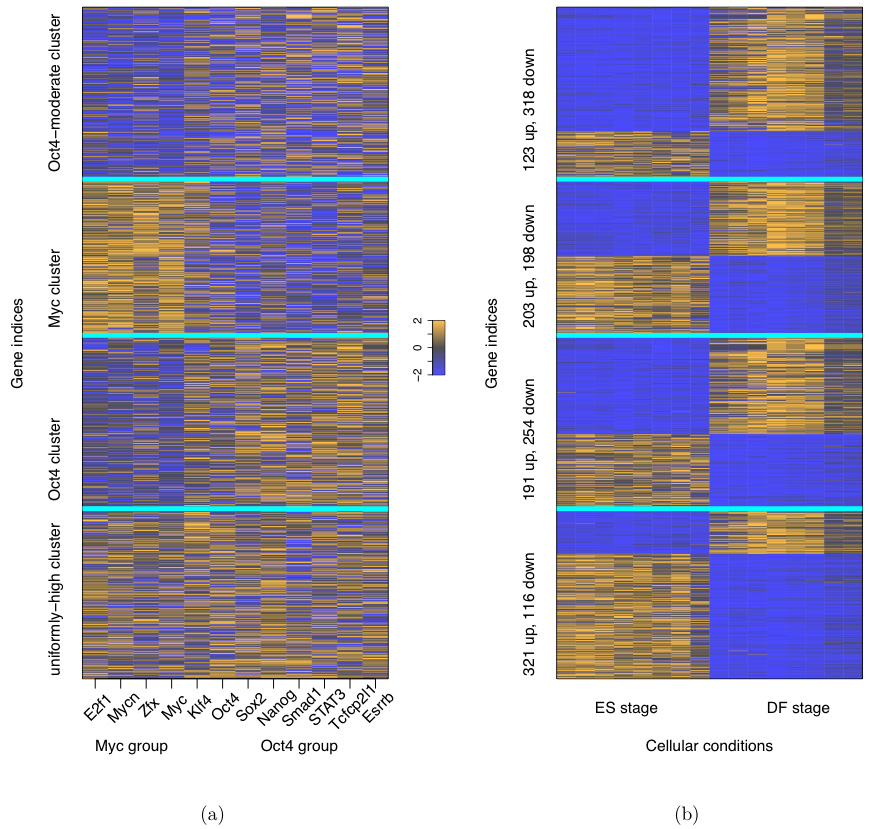
**Association scores and expression profiles with genes aligned between (a) and (b)**. (a) Association scores of the ES genes that are in the uniformly-high cluster, the Oct4 cluster, the Myc cluster, and the Oct4-moderate cluster, with cyan-colored horizontal lines indicating boundaries between the clusters. (b) Gene expression profiles with the first 8 conditions corresponding to ES stage and the rest corresponding to DF stage, annotated by the number of ES-up (indicated by "up") genes and ES-down ("down") genes in each cluster. Note that genes within a cluster are sorted according to their expression fold change. Association scores and gene expression profiles are the same as the ones generated in their original papers. Specifically, the association scores are log-transformed and quantile-normalized [14]; the gene expression profiles in [16] are normalized by the invariant set method implemented by dChip [37]. For display purpose, both the association scores and the gene expression profiles are rescaled to the range between -2 and 2.

Figure 3 shows the association scores of ES genes in the remaining four clusters and their corresponding expression profiles (see Additional file 1 for visualization of two components of association scores: ChIP-seq intensities and distances to TSS). Although the genes in a cluster share a similar binding pattern, a substantial number of these genes are either up- or down-regulated at ES stage. For example, the Myc cluster has around the same number of ES-up genes as ES-down genes. We hypothesize that cofactor binding may help account for the two distinct expression patterns in the same binding cluster. For the Oct4 cluster and the Oct4-moderate cluster, we hope to find cofactors collaborating with the members of the Oct4 group--note that the Oct4 group has stronger association than the Myc group in these two clusters--and thus, the TFs in the Oct4 group are called the *main factors of interest *for these two clusters; analogously, for the Myc cluster, the members of the Myc group are the main factors of interest; for the uniformly-high cluster, all the 12 TFs are the main factors of interest.

Meanwhile, we focus on ES genes in these four clusters (see Additional file 2). Our goal is to search for cofactors whose co-binding (with corresponding main factors of interest) to ES-up genes and to ES-down genes in a cluster are significantly different from each other, and can optimally classify the genes within the cluster.

### Features and feature scores

A *feature *is defined as association between genes and a combination of *w *main factors of interest and one cofactor. Such a combination is referred to as a TF combination. We consider three types of features corresponding to *w *= 2, 1 and 0: A pair of main factors and a cofactor, a single main factor and a cofactor, and a cofactor alone. Whereas a cofactor prefers to co-bind to DNA sequences with two or one specific main factor for the first two types of features, respectively, the last type does not indicate such preferences. Depending on main factors of interest in a cluster, features involving TF combinations constructed in different clusters are different. For example, for the Myc cluster, a TF combination consists of one or two of the main factors of the Myc group (E2f1, Mycn, Zfx, and Myc) and one cofactor. Thus, different clusters have different numbers of features. Given 202 pre-selected potential cofactors (see Methods for selection criteria and Additional file 3 for pre-selected cofactors), there are 13332 $\left(=\left({}_{2}^{12}\right)\times 202\right)$ features with *w *= 2 and 2424 features with *w *= 1 for the uniformly-high cluster. For the Oct4 and Oct4-moderate clusters, there are 4242 and 1414 features corresponding to *w *= 2 and *w *= 1, respectively. For the Myc cluster, there are 1212 features with *w *= 2 and 808 features with *w *= 1. Finally, there are 202 features involving only cofactors (*w *= 0) for all the clusters.

To locate places where a co-binding of a TF combination may happen, we identified genomic regions that contain at least two binding sites of main factors of interest in the ChIP-seq data. Such a genomic region is called a *neighborhood*. Please see Methods for neighborhood construction and Additional files 4, 5, 6 for neighborhood summary statistics such as their lengths and distances to TSS's. As mentioned above, for different clusters, features are different depending on different main factors of interest, and thus different subsets of the ChIP-seq data were used for neighborhood construction. For example, the ChIP-seq data of E2f1, Mycn, Zfx, and Myc were used for the Myc cluster. We then scanned the neighborhoods with the 202 pre-selected TF motifs from TRANSFAC 12.1 [18] and de novo motif discovery to detect possible binding sites of these potential cofactors (see Methods).

Next, a neighborhood is associated with a gene if its center is within *d *bps of the gene's TSS (See Methods for how to specify *d*). Given a gene and its associated neighborhoods containing binding sites of main factors and a cofactor involved in a TF combination, we computed a *feature score *to quantify the association strength between the TF combination and the gene (see Methods). Intuitively, a greater feature score is due to a shorter distance from the location of the TF combination to the TSS and/or a stronger binding strength of the combination.

If a TF combination cannot be associated with a gene because it does not occur in any associated neighborhood of the gene, we say that the feature score between this combination and the gene does not exist (see Additional files 7, 8, 9, 10, 11, 12 for all feature scores).

### Feature significance

For each feature, we employed two-sample comparisons to test whether the feature score distribution of ES-up genes is significantly different from the distribution of ES-down genes. Specifically, the Wilcoxon rank-sum test was utilized to test whether the feature scores in one gene set are significantly greater than the feature scores in the other (due to non-normality of feature score distributions, the nonparametric test was employed); two-sample proportion test was used to test whether the proportion of the genes associated with a TF combination (involved in the feature) in one gene set is significantly greater than that in the other.

For each type of features, many features lead to a multiple testing problem for each kind of test. (Note that a feature type is defined by *w *= 2, 1, 0, the number of main factors in a TF combination as presented in the last section. For example, features involving two main factors are one type of features.) We collected significant features under an FDR cutoff of 10%. We define an *ES-up feature *as a feature that shows a higher proportion in ES-up genes than that in ES-down genes or has significantly higher scores in ES-up genes than ES-down genes. An *ES-down feature *is defined in an analogous way. Table 1 summarizes the number of significant features in each cluster (see Additional file 13 for significant features). For the Oct4 cluster, there are only a few significant features with *w *= 0 but many more significant features with *w *= 1 or 2. For the Myc cluster and the Oct4-moderate cluster, there are also more significant features involving a TF combination than features involving only a cofactor. The result demonstrates that we would have ignored important features if we only considered cofactors alone, and that the constructed features are effective to capture a cofactor's preference for co-binding with main factors for potential gene regulation.

**Table 1:** Numbers of ES-up (U) and ES-down (D) features (FDR < 10%) in different clusters

	*w *= 2		*w *= 1		*w *= 0	
	U	D	U	D	U	D
uniformly-high cluster	2	0	4	3	1	14
Oct4 cluster	7	186	22	10	8	2
Myc cluster	3	76	7	141	0	63
Oct4-moderate cluster	464	0	366	0	102	0

Note: *w *denotes the number of main factors, indicating feature types.

Interestingly, almost all significant ES-up features were discovered by the rank-sum test. This fact may suggest activation of genes at ES stage is more likely to happen when a TF combination is located closer to TSS's, and/or it has a stronger binding strength. In contrast, all significant ES-down features were identified by two-sample proportion test. This phenomenon indicates that the occurrence of a particular TF combination may be sufficient to increase the chance of down-regulation at ES stage--neither its location nor its strength makes differences. Since utilizing features in continuous and binary fashions informs us different meanings, both are useful and should be considered together.

We can reason regulatory roles of a cofactor involved in a significant feature by checking its fold change in the expression profiles and checking whether the feature is an ES-up or ES-down feature. Fold change in the expression profiles is said to be *positive *if the ratio of the mean expression at ES stage over the mean expression at DF stage > 2, and *negative *if the ratio < 0.5. By its definition, an ES-up feature is significantly associated with genes that are up-regulated at ES-stage (and down-regulated at DF stage). Therefore, if a cofactor involved in an ES-up feature shows positive fold change, it may play an activator role in ES cells. On the other hand, if the cofactor is active at DF stage, suggested by its negative fold change, it may repress genes at DF stage. For a cofactor with neither positive fold change nor negative fold change, it may show *uniformly high *expression, defined by average expression index > 500 at both ES and DF stages, and thus may function at both stages. In this case, we may not tell its role as an activator at ES stage from an repressor at DF stage. Following the same logic, we can reason the roles of a cofactor involved in an ES-down feature as either a repressor at ES stage and/or an activator at DF stage. Table 2 summarizes the above reasoning.

**Table 2:** Reasoned regulatory roles of a cofactor

Cofactor expression pattern	ES-up feature	ES-down feature
Positive fold change	ES activator (EA)	ES repressor (ER)
Negative fold change	DF repressor (DR)	DF activator (DA)
Uniformly high	EA and/or DR	ER and/or DA

### Feature selection

Based on the significant results we obtained, we further selected a small subset of features with gene classification, where top features ranked by their significance (according to the tests described in the previous section) were treated as predictors in a naïve Bayes (NB) classifier and ES-up and ES-down genes as two classes (see Methods).

**Figure 4:**
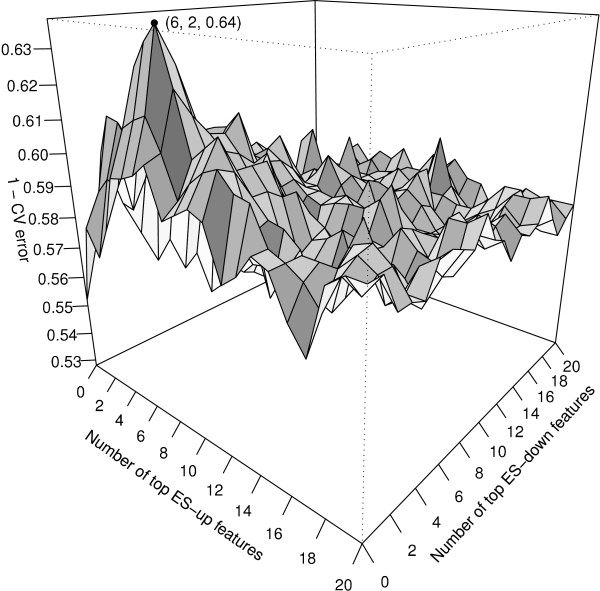
**Ten-fold CV accuracy with different numbers of ES-up and -down features**. Ten-fold CV accuracy with top *k*_1 _ES-up features involving *w *= 1 main factor and top *k*_2 _ES-down features involving *w *= 2 main factors for the Oct4 cluster, 0 ≤ *k*_1_,*k*_2 _≤ 20. The highest CV accuracy 64% is labeled in the figure, involving top 6 ES-up features and top 2 ES-down features.

Since ES-up and ES-down features indicate different biological meanings, we hoped to incorporate information from both ES-up and ES-down features into predictors. To reduce potential combinations of three types of ES-up features (corresponding to *w *= 2, 1, 0) and three types of ES-down features as indicated by six columns in Table 1, we restricted our search strategy to combining only one type of ES-up features and only one type of ES-down features, and thus there are a total of 9 (= 3 × 3) combinations to be examined. Specifically, we used top *k*_1 _ES-up features (of one type) ranked by their *P*-values and top *k*_2 _ES-down features (of one type) as *k *(= *k*_1 _+*k*_2_) predictors in an NB classifier. Figure 4 illustrates the situation where we examined a combination of one type of ES-up features (*w *= 1) and one type of ES-down features (*w *= 2) based on ten-fold cross-validation (CV). It shows that the classification accuracy for enumeration of the two types of features for the Oct4 cluster, where on average the combination of top six ES-up features and top two ES-down features achieves the highest accuracy (minimum CV error). The decreasing trend of the accuracy can also be observed as *k*_1 _and *k*_2 _increase from six and two, respectively. Similar enumeration was conducted for the remaining eight combinations of feature types, and the combination with the minimum CV error was selected. Please see Methods for details of the feature enumeration procedure we employed given two types of features.

Table 3 compares NB classifiers based on only main factors (and thus using association scores) with those utilizing both main factors and selected features involving cofactors. Although little improvement was made for the uniformly-high cluster and the Oct4-moderate cluster, incorporating cofactors can reduce 20% and 11% of CV errors compared to only using main factor effects for the Oct4 cluster and the Myc cluster, respectively. Ouyang et al. [14] adopted the CART algorithm on three principal components of association scores of main factors for gene classification. As a comparison, we also classified ES-up and ES-down genes with the CART algorithm based on the same principal components. This approach showed similar performance to the NB classifiers using only main factors as predictors (Table 3). Again, it was about 16% worse than the classifiers utilizing cofactor effects for the Oct4 cluster and the Myc cluster.

**Table 3:** Ten-fold CV errors and corresponding standard errors

Method	uniformly-high cluster	Oct4 cluster	Myc cluster	Oct4-moderate cluster
NB_MF	0.27 (0.02)	0.45 (0.02)	0.42 (0.02)	0.29 (0.02)
CART	0.27 (0.02)	0.43 (0.02)	0.44 (0.02)	0.28 (0.02)
NB	0.26 (0.02)	0.36 (0.02)	0.37 (0.02)	0.29 (0.02)
(NB_MF - NB)/NB_MF	0.04	0.20	0.11	0.00
(CART - NB)/CART	0.04	0.16	0.16	-0.04

Note: NB_MF--classification with NB classifiers based on only main factors; CART--classification with CART based on three principal components of association scores of main factors as in [14]; NB--classification with NB classifiers utilizing both main factors and cofactors.

There is little improvement by utilizing cofactor information in the uniformly-high cluster and the Oct4-moderate cluster. It may be due to more evident imbalance between ES-up and ES-down genes in each of the two clusters than the other two clusters--73% ES-up genes in the uniformly-high cluster and 72% ES-down genes in the Oct4-moderate cluster. The dominant gene sets may suggest that gene clustering based on the association scores (with only main factor information) already partitions the genes well for these two clusters so that the contribution from features involving cofactors is marginal for further classification.

**Figure 5:**
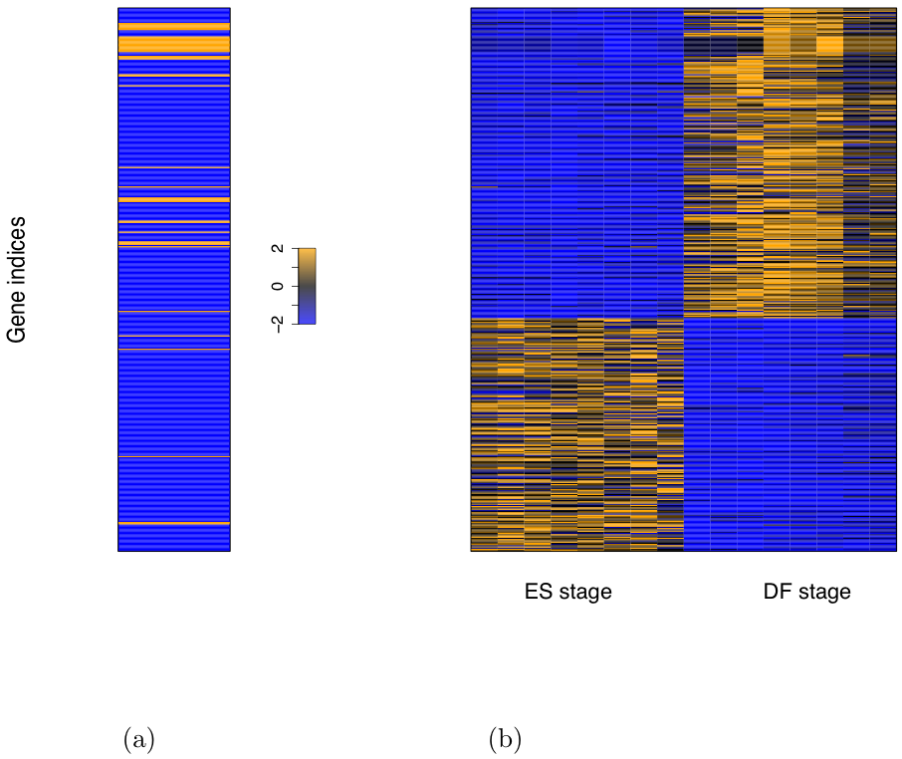
**For the ES-down feature Smad1.STAT3.M00052_NFKB, association and expression profiles (rescaled for display purpose) with genes aligned between (a) and (b) in the Oct4 cluster, with the same order in Figure 3**. (a) Binary association of the feature with the ES genes, with value 2 indicating existence of association and value -2 indicating no association. (b) Gene expression profiles with the first 8 conditions corresponding to ES stage and the rest corresponding to DF stage. Similar to Figure 3, the gene expression profiles are normalized by the invariant set method and rescaled to [-2, 2].

**Figure 6:**
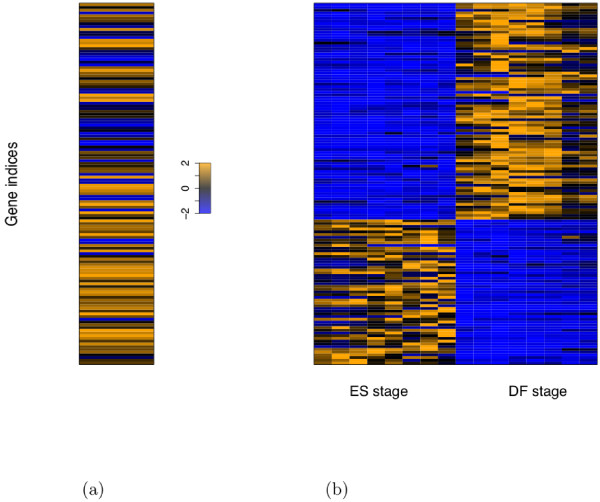
**For the ES-up feature Oct4.M00801_CREB_Q3, feature scores and expression profiles (rescaled for display purpose) with genes aligned between (a) and (b) in the Oct4 cluster, with the same order in Figure 3**. (a) Feature scores for the ES genes. (b) Gene expression profiles with the first 8 conditions corresponding to ES stage and the rest corresponding to DF stage.

Table 4 shows selected features based on the best CV classification results. We denote a feature by the concatenation of main factor names and the motif name of a cofactor with dot as separator. A motif name starting with M is from TRANSFAC, and with N from de novo discovery. For example, the feature Smad1.STAT3.M00052_NFKB involves the main factors Smad1 and STAT3 and a cofactor whose motif name is M00052_NFKB in TRANSFAC. Figure 5 visualizes how this selected feature helps differentiate the ES-down and ES-up genes in the Oct4 cluster. The proportion of the ES-down genes associated with this feature is much higher than that of the ES-up genes. Therefore, this features is defined as an ES-down feature. On the other hand, Figure 6 shows that the feature Oct4.M00801_CREB_Q3 has higher feature scores for the ES-up gene set than the ES-down gene set, thus making this feature an ES-up feature.

**Table 4:** Summary of selected features

Cluster	ES U/D	Feature	Locuslink	Cofactor	FC	Role	*P*-value	LS
UH	U	E2f1.M00233_MEF2_04	17258	Mef2a	N	DR	8.66E-07	
UH	U	Sox2.M00974_SMAD _Q6_01	17126	Smad2	N	DR	1.03E-04	
UH	U	Sox2.M00974_SMAD_ Q6_01	17128	Smad4	H	EA/DR	1.03E-04	[38]
UH	U	Esrrb.M00971_ETS_Q6	13709	Elf1	N	DR	1.25E-04	
UH	U	Esrrb.M00971_ETS_Q6	69257	Elf2	N	DR	1.25E-04	
UH	U	Esrrb.M00971_ETS_Q6	14390	Gabpa	P	EA	1.25E-04	

Oct4	D	Smad1.STAT3.M00052_NFKB	19697	Rela	N	DA	2.66E-07	[19]
Oct4	D	Smad1.STAT3.M00415_AREB6_04	21417	Zeb1	N	DA	1.51E-06	[20]
Oct4	U	Tcfcp2l1.M00771_ETS_Q4	69257	Elf2	N	DR	1.82E-06	
Oct4	U	Tcfcp2l1.M00771_ETS_Q4	13709	Elf1	N	DR	1.82E-06	
Oct4	U	STAT3.M00251_XBP1_01	22433	Xbp1	N	DR	1.37E-05	
Oct4	U	Nanog.M00462_GATA6_01	14465	Gata6	N	DR	2.69E-05	[21]
Oct4	U	Nanog.M00134_HNF4_01	15378	Hnf4a	N	DR	1.05E-04	
Oct4	U	Esrrb.N00007_SoxOctComp	18999	Sox2 & Oct4	P	EA	1.32E-04	
Oct4	U	Oct4.M00801_CREB_Q3	11911	Atf2	H	EA/DR	1.66E-04	
Oct4	U	Oct4.M00801_CREB_Q3	11908	Atf1	H	EA/DR	1.66E-04	
Oct4	U	Oct4.M00801_CREB_Q3	11910	Atf7	N	DR	1.66E-04	

Myc	D	M00233_MEF2_04	17258	Mef2a	N	DA	3.83E-05	
Myc	D	M00971_ETS_Q6	14390	Gabpa	P	ER	4.92E-05	
Myc	D	M00971_ETS_Q6	69257	Elf2	N	DA	4.92E-05	
Myc	D	M00971_ETS_Q6	13709	Elf1	N	DA	4.92E-05	
Myc	D	N00003_Otx2	18424	Otx2	P	ER	1.05E-04	[22,23]
Myc	D	M00034_P53_01	22059	Trp53	P	ER	1.61E-04	[24]
Myc	D	M00421_MEIS1BHOXA9_02	17268	Meis1	N	DA	1.95E-04	
Myc	D	M00512_PPARG_01	19016	Pparg	N	DA	3.64E-04	[25]
Myc	D	N00009_Klf4	16600	Klf4	N	DA	3.97E-04	[26]
Myc	D	N00009_Klf4	16598	Klf2	P	ER	3.97E-04	[26]
Myc	D	N00009_Klf4	12224	Klf5	H	ER/DA	3.97E-04	[26]
Myc	D	M00764_HNF4_DR1_Q3	15378	Hnf4a	N	DA	8.46E-04	
Myc	D	M00422_FOXJ2_01	60611	Foxj2	N	DA	9.43E-04	
Myc	D	M00724_HNF3ALPHA_Q6	15375	Foxa1	N	DA	9.81E-04	
Myc	D	M01011_HNF1_Q6_01	21410	Hnf1b	N	DA	1.16E-03	
Myc	U	Myc.M00480_LUN1_01	106021	Topors	H	EA/DR	1.43E-04	
Myc	U	Mycn.M00931_SP1_Q6_01	20683	Sp1	H	EA/DR	1.69E-04	
Myc	U	Mycn.N00017_hB_Sall4	99377	Sall4	H	EA/DR	1.81E-04	[39]

Oct4-M	U	M01111_RBPJK_Q4	19664	Rbpj	P	EA	6.51E-06	[28,29]
Oct4-M	U	M00450_ZIC3_01	22773	Zic3	P	EA	1.74E-05	[30]
Oct4-M	U	N00001_Sox2	20674	Sox2	P	EA	2.45E-04	[3]
Oct4-M	U	M00225_STAT3_01	20848	Stat3	N	DR	2.50E-04	[6]

Note: UH cluster is shorthand for the uniformly-high cluster; Oct4-M cluster is shorthand for the Oct4-moderate cluster. The column "ES U/D" indicates whether a feature is an ES-up feature (U) or an ES-down feature (D). Locuslinks, cofactor gene names, and fold change (FC) of gene expression (N--negative fold change, P--positive fold change, H--uniformly high) of cofactors are listed correspondingly. The column "LS" indicates related literature support for a feature.

Although features involving two main factors and one cofactor are not selected for other clusters, the two ES-down features for the Oct4 cluster are of this type. In addition, except the Oct4-moderate cluster, there are features involving one main factor and one cofactor in all the other clusters. Not only these features show strong statistical significance (with FDR < 10%), but also they are useful from the classification perspective. The evidence from these aspects suggests that the cofactors involved in these features are likely to be regulators in ES cells.

### Literature support

For more than one third of the features in Table 4, existing literatures provide supporting evidence of related biological functions. We list relevant literatures for specific features in the last column of the table. Here we present support for some features in detail. Reported in the Oct4 cluster, Rela, which binds to M00052_NFKB sites, was inferred to co-bind with STAT3 and Smad1 for activating genes at DF stage. Lee et al. [19] suggested that Rela, p300, and STAT3 are in the same DNA-binding complex in tumors. In addition, they provided related references showing that Rela and STAT3 stimulate a highly overlapping repertoire of prosurvival, proliferative, and proangiogenic genes. The above findings show that the collaboration exists between STAT3 and Rela, and our analyses further reveal that there is a need for these two TFs to work with Smad1 in order to regulate genes in ES cells (note that the feature STAT3.M00052_NFKB only shows marginal significance with *P-*value = 0.06). The feature Smad1.STAT3.M00415_AREB6_04 involves Zeb1, which binds to M00415_AREB6_04 sites, and Smad1, which is a receptor-regulated Smad (R-Smad) and a key component of the BMP signaling pathway [7]. Consistently, Postigo et al. [20] found that Zeb1 synergizes with Smad-mediated transcriptional activation and regulates BMP signaling, and that R-Smad and Zeb1 form a complex that recruits p300 much more efficiently, thus accounting for their transcriptional synergy. These results support Smad1 and M00415_AREB6_04 in the identified regulatory code, but STAT3 is necessary for them to receive special attention for ES cell studies--the significance of the feature Smad1.M00415_AREB6_04 (with *P-*value = 0.02) is not as strong as the reported three-TF feature (with *P-*value = 1.51 × 10^-6^).

According to Gata6's inferred role from the feature Nanog.M00462_GATA6_01--a repressor at DF stage, it may repress genes activated by Nanog at ES stage. This exemplifies the situation where a main factor and a cofactor in an identified TF combination may not always work with each other at the same time. The above reasoning is in line with one of the findings in [21], which demonstrated that antagonism between Nanog and Gata6 leads to segregation of epiblast and primitive endoderm within inner cell mass, and that an excess of Gata6 pushes the cell into the endoderm lineage.

We now discuss some studies on cofactors identified for the Myc cluster. Consistent with the inferred role of Otx2 as a repressor at ES stage, the results in [22] revealed that Otx2 regulates neuronal progenitor domains by repressing Nkx2.2 in the ventral midbrain. In another study [23], Puelles et al. suggested that Otx2 represses GABAergic differentiation to control glutamatergic progenitors of the thalamus. Another predicted repressor at ES stage, Trp53, induces differentiation of mouse ES cells via suppressing Nanog expression [24]. We reasoned that Pparg activates genes at DF stage. Indeed, it is one of the peroxisome proliferator-activated receptors (PPARs), a group of three nuclear receptor isoforms interacting with other factors to increase transcription initiation rate [25].

Although Klf4 is dispensable for maintenance of self-renewal and pluripotency of ES cells, concurrent depletion of Klf2, Klf4, and Klf5 leads to ES cell differentiation [26]. Hall et al. [27] showed that Oct4 mainly induces Klf2 and LIF/STAT3 selectively enhances Klf4 expression. Our study revealed that the binding sites of these Klfs are preferentially enriched in the neighborhoods associated with the ES-down genes in the Myc cluster, suggesting possible cooperation between the Oct4 group and the Myc group via Klfs on gene repression in ES cells--Oct4/STAT3 may introduce Klfs that cooperate with the members of the Myc group.

In tune with Rbpj's reasoned role as an activator from the feature M01111_RBPJK_Q4 discovered in the Oct4-moderate cluster, the intracellular part of the cell surface Notch1 receptor (Notch1-IC) alters the function of Rbpjκ to be a transcription activator [28]. Additionally, Robert-Moreno et al. [29] showed that activation of Gata2 expression by Notch1/Rbpjκ is essential for the onset of definitive hematopoiesis in the mouse embryo. Our analysis suggested that Zic3 may be a transcriptional regulator at ES stage. Lim et al. [30] established that repression of Zic3 in ES cells induces expression of several markers of the endodermal lineage and leads to significant reduction of Nanog expression, and thus Zic3 plays an important role in the maintenance of pluripotency by preventing endodermal lineage specification in ES cells. Zic3 may activate repressors of the endoderm markers and thus works as an activator in ES cells. Given the above evidence from the existing literatures, other identified cofactors may also play some unknown roles at ES/DF stages, and thus may be worth follow-up experimental verification.

### Support by target prediction

Based on the formulation of an NB classifier, we predicted target genes that are regulated by TFs involved in a feature. The prediction is through checking the change in the probability ratio for classification after a feature is excluded from predictors of a classifier that employs the selected features (see Methods). To provide evidence for predicted targets, we checked gene expression profiles of identified cofactors and their predicted targets in another data set--the RAi (retinoic-acid-induced) data [9]. Gene expression was profiled for 6 days when ES cells underwent retinoic-acid-induced differentiation. We treated day 0 as ES stage and day 4-6 as DF stage, and computed fold change of the expression profile of day 0 over the mean expression of day 4-6 as in [16]. We then checked whether fold change of an identified cofactor in the RAi data matches its fold change in our study. Besides exact match, we allowed uniformly high status in one data set to match positive/negative fold change in the other because the status indicates potential functioning of a cofactor at both stages.

If a match is found, expression change of predicted targets is checked for validation: According to our analysis, it is expected that expression of targets of an activator at ES stage and/or a repressor at DF stage (in terms of a cofactor's regulatory role) should decrease after differentiation, and conversely, expression of targets of a repressor at ES stage and/or an activator at DF stage should increase. The proportion of targets whose observed expression satisfied the above expectation is high, ranging from 61% to 92%, as shown by *Pc* in Table 5, where selected quantiles of targets' fold change for each feature are also presented for reference. Based on this consistent result, the target-prediction practice further supports the identified cofactors and their regulatory roles.

**Table 5:** Summary of expression fold change of predicted targets in the RAi data

Cluster	Feature	Cofactor	FC_RAi	*N*_*b*_	*N*_*e*_	*P*_*c*_	1Q	Median	3Q
UH	E2f1.M00233_MEF2_04	Mef2a	N	198	191	0.90	1.67	2.28	4.64
UH	Sox2.M00974_SMAD_Q6_01	Smad2	H	92	90	0.89	1.82	3.01	8.50
UH	Sox2.M00974_SMAD_Q6_01	Smad4	H	92	90	0.89	1.82	3.01	8.50
UH	Esrrb.M00971_ETS_Q6	Elf2	H	150	144	0.92	1.62	2.32	4.12
UH	Esrrb.M00971_ETS_Q6	Gabpa	H	150	144	0.92	1.62	2.32	4.12

Oct4	Smad1.STAT3.M00052_NFKB	Rela	H	39	29	0.72	0.99	1.97	2.65
Oct4	Smad1.STAT3.M00415_ AREB6_04	Zeb1	N	36	25	0.76	1.16	1.83	2.65
Oct4	Tcfcp2l1.M00771_ETS_Q4	Elf2	H	90	82	0.82	1.16	1.97	4.15
Oct4	STAT3.M00251_XBP1_01	Xbp1	H	23	23	0.78	1.16	1.75	3.86
Oct4	Nanog.M00462_GATA6_01	Gata6	N	51	48	0.83	1.29	2.10	4.20
Oct4	Esrrb.N00007_SoxOctComp	Sox2 & Oct4	P	108	101	0.82	1.14	1.95	4.04
Oct4	Oct4.M00801_CREB_Q3	Atf1	P	45	40	0.75	1.00	1.91	4.41

Myc	M00233_MEF2_04	Mef2a	N	170	163	0.90	1.33	1.66	2.53
Myc	M00971_ETS_Q6	Gabpa	H	173	167	0.89	1.33	1.70	2.67
Myc	M00971_ETS_Q6	Elf2	H	173	167	0.89	1.33	1.70	2.67
Myc	N00003_Otx2	Otx2	P	166	160	0.90	1.34	1.67	2.59
Myc	M00034_P53_01	Trp53	P	166	160	0.91	1.34	1.67	2.71
Myc	M00512_PPARG_01	Pparg	N	167	160	0.89	1.34	1.70	2.59
Myc	N00009_Klf4	Klf2	P	184	177	0.89	1.33	1.68	2.58
Myc	N00009_Klf4	Klf5	P	184	177	0.89	1.33	1.68	2.58
Myc	M00422_FOX J2_01	Foxj2	N	168	161	0.90	1.34	1.70	2.58
Myc	M00724_HNF3ALPHA_Q6	Foxa1	N	177	170	0.89	1.32	1.67	2.46
Myc	M01011_HNF1_Q6_01	Hnf1b	N	180	174	0.89	1.34	1.67	2.48
Myc	Myc.M00480_LUN101	Topors	H	73	69	0.81	1.15	1.60	2.26
Myc	Mycn.M00931_SP1Q6_01	Sp1	H	80	78	0.73	0.95	1.43	2.16
Myc	Mycn.N00017_hB_Sall4	Sall4	P	87	82	0.74	0.98	1.57	2.60

Oct4-M	M01111_RBPJK_Q4	Rbpj	P	62	57	0.61	0.70	1.27	2.23
Oct4-M	M00450_ZIC3_01	Zic3	P	43	41	0.63	0.80	1.28	2.23
Oct4-M	N00001_Sox2	Sox2	P	49	46	0.57	0.70	1.09	1.45
Oct4-M	M00225_STAT3_01	Stat3	H	44	43	0.67	0.84	1.28	1.79

Note: FC_RAi is fold change of cofactor expression. *N*_b _is the number of predicted targets for a feature. *N*_*e *_is the number of targets having expression available in [9]. *P*_*c *_is the proportion of *N*_*e *_genes with expected expression change. 1Q, Median, and 3Q are, respectively, the 1st quartile, the median, and the 3rd quartile of targets' fold change, which is inverted for ES-down features for convenient comparison.

Among all the predicted targets, an interesting category is the ones that themselves are main factors or identified cofactors, indicating cascade regulatory pathways. Noticeably, multiple targets in this category are observed for each of the three combinations selected in the uniformly-high cluster (Table 4): Otx2, Klf2, Tcfcp2l1, Pou5f1, and Trp53 are potential targets of E2f1 and Mef2a; Otx2, Pou5f1, Trp53, and Sox2 are possibly regulated by Sox2 and Smad4 or Smad2; Klf2, Tcfcp2l1, Pou5f1, Trp53, and Sox2 are among the predicted targets of Esrrb and Elf1 or Elf2 or Gabpa. Other clusters do not show this phenomenon. It implies that genes in the uniformly-high cluster (that is, genes highly associated with all the 12 main factors) may play important roles in forming complex network structure and should receive particular attention when modeling regulatory networks is the goal of a study. Consistently, the three related top GO terms enriched in the targets of this cluster are: Negative regulation of biological process, regulation of developmental process, and regulation of cell differentiation.

We summarize biological implications of our results as follows: Based on the definitions of ES-up/-down features, we inferred the regulatory roles of the potential cofactors involved in the reported features (Table 4), some of which are supported by relevant literatures; the gene-classification practice shows that the identified TF combinations may explain the gene expression; the expected expression change of the predicted targets in another data set adds more confidence in the inferred regulators' function. The results of this study can provide new clues to expand the core regulatory network among main factors and to identify novel combinatorial regulation of the rich expression profiles in mouse ES cells.

## Discussion

Although the classification improvement of our approach for the Oct4 cluster and the Myc cluster comparing to the other methods in Table 3 is substantial and encouraging, the absolute CV errors are quite high. Such results could be due to the following reasons. First, as shown in Table 1, there are more significant features than what has been enumerated under our search strategy. Some of them are biologically meaningful in explaining gene expression, and thus have the potential to further reduce classification errors. For example, the neuronal repressor REST is involved in the ES-down feature Oct4.Smad1.M00325_NRSE_B (with *P-*value = 2.07 × 10-^5^) for the Oct4 cluster, which suggests REST collaborating with the key TFs Oct4 and Smad1 for gene down-regulation at ES stage. In line with its repressor role, this cofactor has been shown to maintain self-renewal and pluripotency in mouse ES cells through suppression of the microRNA miR-21 [31]. Thus, this ES-down feature may be worth further investigation. However, one challenging question is how features like this can be discovered; simply increasing the number of top features in NB classifiers may include many noisy features and thus degrade classification performance according to our experience. Therefore, procedures with stronger selective power are needed. Second, sophisticated learning methods such as boosting and Bayesian additive regression trees (BART) may have better classification performance (as Zhou and Liu [32] demonstrated in TF-DNA binding problems), but these methods may have difficulties in interpretation of their results. In the framework we employed, not only do the statistical tests help reduce search space by focusing on significant features for feature selection in classification, but they also provide ground for biologically meaningful interpretations of features as we have discussed in the section of feature significance. Third, although in this study we focused on TF regulatory control, TF binding is only one way of gene regulation. Other mechanisms such as DNA methylation are also involved in regulating gene expression. Lack of their information may lead to low classification accuracy. Thus, readers should treat our report in this study as intermediate results--further investigation of this challenging classification problem is needed.

In the current framework, we consider features involving only one cofactor and up to two main factors. Further extension to more cofactors and main factors is possible, and the extension may gain additional capacity to differentiate the gene expression patterns. However, with more and more specific TF combinations, the support (that is, genes that are regulated by the involved TFs) may become less and less, and thus the gained capacity may not be detected. On the other hand, the extension will lead to combinatorial explosion. For example, including one more cofactor will bring ~100 times more features into consideration. This will result in much more tests for each feature type, which may cause high FDR for the current data size. In addition, following the logic of combinatorial regulatory codes, another kind of features would be a pair of main factors without considering a cofactor in our framework. Alternatively, one could treat one main factor as a cofactor of the other main factor. We explored this possibility and found that little improvement can be made in gene classification. This may be because classification happens within each gene cluster and collaborative efforts among main factors have already been captured by clustering.

Besides ES genes, other genes with less substantial fold change, referred to as *ES-neutral genes*, may also be informative. If the function of a TF combination is only to activate genes in ES cells, we expect that the contrast in the feature between ES-up and ES-down genes may be more significant than the contrast between ES-up genes and ES-neutral genes. The reason is that the TF combination may have a lower chance to randomly occupy regulatory elements of an ES-down gene (and thus would up-regulate it at ES stage) than an ES-neutral gene, which may be activated but has insignificant fold change. Similarly, focusing on ES genes may be helpful for detecting a TF combination as a pure ES repressor. On the other hand, if a particular combination works as either an activator or a repressor in ES cells depending on other cellular context or targets, a feature may then be strongly associated with both ES-up and ES-down genes, leading to insignificant test results and defeating detection. In this case, the ES-neutral gene set, containing genes that are not regulated by the combination, may provide a better contrast for detecting such a combination. The detection may be conducted in the same framework with one of ES gene sets replaced by ES-neutral genes. In summary, many possible directions can be explored based on this study.

## Conclusions

This study suggests a list of TF combinations which may play important regulatory roles in ES cells based on computational analyses. They serve as top candidates for experimental evaluation. We provided computational evidence of the finding from three aspects: 1. The features involving identified TF combinations show strong statistical significance; 2. the classifiers based on them have the optimal performance in classifying gene expression and also achieve substantial improvement over classifiers utilizing only main factor information; 3. their predicted target genes (based on classification) in another independent data set show expected fold change as in our prediction. In addition to the above evidence, existing literatures provide support for reasoned regulatory roles of some identified cofactors. In summary, this study effectively reveals combinatorial co-binding patterns which involve potential regulators in mouse ES cells.

## Methods

### Association scores

Suppose that *n *binding sites (peaks) of a main factor are located within 10^6 ^base pairs (bps) of a gene's TSS. The association score between the gene and the main factor is defined by

(1) $$\overset{n}{\underset{i=1}{\sum }}f_{i}e^{-\frac{d_{i}}{d_{0}}},$$

where *f*_i _denotes the ChIP-seq signal intensity on the *i*th main factor binding site, *d*_*i *_represents the distance from the site to the gene's TSS, and *d*_0 _is a weighting constant, controlling the speed of the exponential decay.

### Motif selection

If a TF's expression has small fold change or is very low, the TF may not function at either ES or DF stage. Therefore, for motif scanning, we selected motifs whose corresponding TF's expression is either uniformly high or shows positive/negative fold change. Please refer to Additional file 3 for the list of the selected motifs and their expression fold change.

### Neighborhood construction

With the following procedures, we constructed genomic regions where we searched for combinatorial TF binding sites for gene regulation. First, a genomic neighborhood of a peak (from the ChIP-seq data) is created via extending 500 base pairs (bps) from the peak center coordinate in both directions; then, two neighborhoods are merged into one neighborhood if a peak center in one neighborhood is located within 500 bps of a peak center in the other neighborhood; the merging operation is made recursively till no two neighborhoods can be merged. We focused on neighborhoods which include at least two peaks. The above construction and consideration were inspired by [11] and [12]--they had discovered a good number of genomic locations co-occupied by multiple TFs based on ChIP-seq and bioChIP-chip experiments, respectively. The neighboring peaks indicate that these genomic islands may be potential regulatory regions where co-binding is likely to happen.

### Binding-site scanning procedure

We now describe how we computed motif score of a sequence segment of width *w*--a *w*-mer--and determined whether it is a binding site. Given a sequence *S *of length *L*, we first estimated ψ_*b*_, the transition matrix of a first-order Markov model as a background model. Given the position-specific weight matrix (PWM) for a motif ψ_*m*_, we then calculated the following probability ratio *r *as the motif score for a *w*-mer located at the *i*th position of *S *for *i *= 1,..., *L *- *w *+ 1:

(2) $$r=\frac{P\left(S\left[i,i+w-1\right]|\Psi_{m}\right)}{P\left(S\left[i,i+w-1\right]|\Psi_{b}\right)}.$$

We also scanned the complementary strand of *S *for computing motif scores. Note that motif scanning was applied after repeat sequences were masked out.

To decide whether a motif score is significant, we randomly drew 20 matched control sequences from the mm8 genome using the CisGenome program [33] for a neighborhood sequence. For every control sequence, its length and distance from its center to its closest gene's TSS matched the length of the neighborhood sequence and the distance from the neighborhood center to the TSS of the gene closest to the neighborhood, respectively. The later match is shown to provide a comparative or better match to binding region GC-content than genome-wide controls [34]. Thus, it improves accuracy of binding site detection. We first scanned matched control sequences for a cluster with Oct4 PWM and built a null distribution for the motif scores of Oct4. We then found a cutoff *P*-value 9.09 × 10^-5 ^corresponding to *r *= 1000, which is reasonable according to our past experience. We then collected significant sites as Oct4 binding sites where corresponding *w*-mers' scores are greater than 1000. For other motifs, we first used *P*-value 9.09 × 10^-5 ^to find score cutoffs from null distributions and then recorded binding sites according to the cutoffs.

### Computing feature scores

Let *k *index the associated neighborhoods of a gene. Suppose that *n*_*k *_and *m*_*k *_are the number of main factor binding sites (peaks) and the number of binding sites of a cofactor in the *k*th neighborhood, respectively. The feature score is defined by

(3) $$\sum_{k}\left[(\prod_{i=1}^{n_{k}}f_{ki}){}^{\frac{1}{n_{k}}}{\left(\prod_{j=1}^{m_{k}}g_{kj}\right)}^{\frac{1}{m_{k}}}\right]e^{-\frac{\frac{1}{n_{k}+m_{k}}\left(\sum_{i=1}^{n_{k}}d_{ki}+\sum_{j=1}^{m_{k}}d_{kj}\right)}{d_{0}}},$$

where *f*_*ki *_denotes the ChIP-seq signal intensity on the *i*th main factor binding site, *g*_*kj *_denotes the motif score of the *j*th cofactor binding site, and *d*_*ki *_and *d*_*kj *_represent the distances to the gene's TSS from the two sites, respectively. Similar to the distance limit for allowing a binding site to be associated with a gene in [14], the longest distance for associating a neighborhood with a gene, *d*, is taken to be 10^6^--that is, neighborhoods that are 1 million bps away from the TSS are not considered, and *d*_0 _= 5000 is a constant for controlling the exponential decay. Feature scores are computed and used in natural-log scale.

We can understand a summation term, which corresponds to contribution from the *kth *neighborhood, in (3) by parts: The part in the brackets is a combined binding strength based on respective geometric averages--one component from main factors and the other from a cofactor; it is then exponentially down-weighted according to the average distance from binding sites to the TSS.

**Figure 7:**
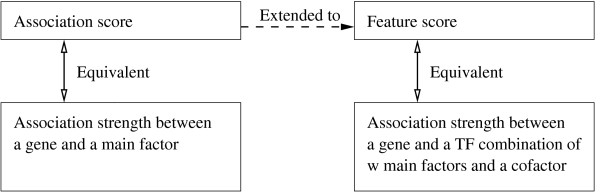
**Concepts of association scores and feature scores and their relationship**. The concepts are briefly defined in the bottom two boxes.

The feature score definition is an extension of the definition of association scores (1). Since an association score is defined for only one main factor with a gene, it concerns only the main factor's ChIP-seq signal intensities and the distances from their binding sites to the TSS. The study [14] showed that in predicting gene expression association scores are superior to traditional scores based on binary association of binding sites with a gene, which do not utilize ChIP-seq signal intensities and relative distances between sites and a gene. In our study, we extend association scores to feature scores for quantifying the association between a TF combination and a gene. To avoid possible confusion about the concepts of association scores and feature scores, Figure 7 summarizes their differences and relationship.

### NB classifier and feature enumeration

Suppose there are *k *predictors in total, denoted by **X **with *X*_*j *_being the *j*th predictor for *j *= 1,..., *k*. Let *Y *denote class label: *Y *= 1 indicates that a gene is an ES-up gene; *Y *= 0 indicates that a gene is an ES-down gene. Let Ψ represent related parameters. An NB classifier is defined by

(4) $$\frac{P(Y=1|\text{X},\Psi )}{P(Y=0|\text{X},\Psi )}=\frac{P(Y=1)\prod_{j=1}^{k}P(X_{j}|\Psi_{j1})}{P(Y=0)\prod_{j=1}^{k}P(X_{j}|\Psi_{j0})}.$$

The classifier compares probabilities that a gene belongs to a class given its predictors, and assigns a class label *y *= argmax _*y*∈{1,0} _*P*(*Y *= *y *| **X**, **Ψ**) to a gene. In the equation, *P*(*Y*) denotes the prior probabilities of the two classes, and *P*(*X*_*j *_| Ψ _j*Y*_) is the conditional density of the *j*th predictor given *Y *(= 1 or 0). The probability of a gene belonging to a class given predictors **X **is computed under the assumption that predictors are conditionally independent of one another given a class label.

In Equation (4), the prior probabilities of the two classes *P*(*Y*) are *p*_1 _and 1- *p*_1 _for *Y *= 1 and 0, respectively. In the density of the *j*th predictor *P*(*X*_*j *_| Ψ _*jY*_), *X*_*j *_is a random variable denoting (1) whether the feature score of the *j*th predictor exists and (2) the feature score if it exists (note that a feature score may not exist for a gene if the feature cannot be associated with the gene, as explained at the end of the section of Feature and feature scores): Let the indicator variable *I*_*j *_= 1 if the feature score of the *j*th predictor exists for a gene, and *I*_*j *_= 0 otherwise; then if *I*_*j *_= 1, *P*(*X*_*j *_| Ψ _*jY*_) = λ_*jY*_*f*_*jY*_(*X*_*j*_), where λ_*jY *_is the probability that a gene with class label *Y *has a feature score of the *j*th predictor, and *f*_*jY *_is the density function of the *j*th predictor's feature score in class *Y*. On the other hand, if the feature of the *j*th predictor cannot be associated with a gene, *X*_*j *_is then reduced to the indicator variable *I*_*j*_, and thus *P*(*X*_*j *_| Ψ _*jY*_) = 1 - λ_*jY *_given *I*_*j *_= 0 in this case. To summarize the above two cases, $P\left(X_{j}|\Psi_{jY}\right)={\left(1-\lambda_{jY}\right)}^{1-I_{j}}{\left(\lambda_{jY}f_{jY}\left(X_{j}\right)\right)}^{I_{j}}.$

The parameters and density functions were estimated from a training data set as follows ${\hat{p}}_{1}=\frac{N_{1}}{N}$, where *N*_1 _is the number of ES-up genes, and *N *is the total number of ES genes. ${\hat{\lambda }}_{jY}=\frac{N_{jY}}{N_{Y}}$, where *N*_*jY*_ is the number of the genes having *X*_*j*_'s scores in class *Y*, and *N*_*Y *_is the number of the genes in class *Y*. We adopted a kernel method to estimate the density function *f*_*jY*_,

$${\hat{f}}_{jY}\left(x\right)=\frac{1}{N_{jY}h_{jY}}\overset{N_{jY}}{\underset{i=1}{\sum }}K\left(\frac{x-x_{jYi}}{h_{jY}}\right),$$

where x _*j*__*y*__*i *_is the score of *X*_*j *_for the *i*th gene in class *Y, K*(·) is the standard normal density, and *h*_*jY *_is the bandwidth of the kernel for *X*_*j *_in class *Y*, calculated according to Silverman's rule of thumb (see page 48 of [35]). Since top features or predictors from two-sample proportion test are based on only binary information, the density function of feature scores *f*_*jY*_(*x*) is excluded from the computation of *P*(*X*_*j *_| Ψ_*jY*_) for these predictors.

Given one type of ES-up features and one type of ES-down features, we enumerated every possible combination of *k*_1 _and *k*_2 _(0 ≤ *k*_1_, *k*_2 _≤ 20) for the above NB classifier formulation. We considered utilizing up to 20 top features due to the following two reasons: 1. Such an option covers 2/3 of the significant cases in Table 1, that is, the number of significant features (of one type) is less than 20 for 16 out of the 24 cases in the table; 2. we observed that CV errors in general increase considerably when *k*_1 _and/or *k*_2 _become large (for example, Figure 4). Predictors involving main factors alone were also included (as control variables), with their association scores utilized in the same way as feature scores. When *k*_1 _= *k*_2 _= 0, these are the only predictors. Minimum CV errors were used to determine the number of features *k*_1 _and *k*_2_.

### Target prediction

A gene is predicted to be regulated by a TF combination involved in an ES-up (-down) feature if this gene satisfies the following two conditions: 1. It is an ES-up (-down) gene; 2. after the feature is excluded from predictors of an NB classifier, we observe a *decrease *(an *increase) *in the ratio of the probability of the gene as an ES-up gene over that as an ES-down gene with the remaining predictors (at the left-hand side of Equation (4)). The first condition ensures that target prediction be consistent with ES-up/-down feature definitions, which are based on statistical significance of one-sided test results; the operation in the second condition intends to mimic knockout of a TF combination and to check expected expression change of potential targets. Additional file 14 lists predicted targets ranked by fold change in the probability ratio. Readers can refer to Additional file 15 for different Gene Ontology (GO) terms, which were retrieved by the DAVID program [36], enriched in the targets of different clusters to learn about their biological functions.

## Competing interests

The authors declare that they have no competing interests.

## Authors' contributions

GC and QZ conceived of the study; GC analyzed data and wrote the paper; both authors revised and proofread the manuscript; all authors read and approved the final manuscript.

## Supplementary Material

Additional file 1 **Association score components**. Association score components (rescaled for display purpose) with genes aligned between (a) and (b) as in Figure 3. (a) Sum of the ChIP-seq intensities: $\sum_{i=1}^{n}f_{i}$ (see Formula (1)) (b) Sum of the exponentiated negative distances to TSS: $\sum_{i=1}^{n}e^{-\frac{d_{i}}{d_{0}}}.$ Click here for file

Additional file 2 **ES gene sets**. This table lists ES genes in different clusters. Click here for file

Additional file 3 **Pre-selected motifs**. This table lists pre-selected motifs. Click here for file

Additional file 4 **Number of ChIP-seq binding sites of main factors of interest in neighborhoods**. This table lists the number of ChIP-seq binding sites of main factors of interest in neighborhoods for each cluster. Click here for file

Additional file 5 **Summary of neighborhood lengths**. This table summarizes neighborhood lengths (in bps). Click here for file

Additional file 6 **Percentiles of distances from neighborhood centers to TSS's**. This table summarizes distances (in bps) from neighborhood centers to TSS's. Click here for file

Additional file 7 **Feature scores for the uniformly-high cluster (a)**. This table contains first 1/3 of feature scores (in log scale) for the uniformly-high cluster, with NA indicating nonexistence of scores. Additional files 7, 8 and 9 should be combined (or concatenated into one file) to get all feature scores for the uniformly-high cluster. Click here for file

Additional file 8 **Feature scores for the uniformly-high cluster (b)**. This table contains second 1/3 of feature scores (in log scale) for the uniformly-high cluster. Click here for file

Additional file 9 **Feature scores for the uniformly-high cluster (c)**. This table contains third 1/3 of feature scores (in log scale) for the uniformly-high cluster. Click here for file

Additional file 10 **Feature scores for the Oct4 cluster**. This table contains feature scores (in log scale) for the Oct4 cluster. Click here for file

Additional file 11 **Feature scores for the Myc cluster**. This table contains feature scores (in log scale) for the Myc cluster. Click here for file

Additional file 12 **Feature scores for the Oct4-moderate cluster**. This table contains feature scores (in log scale) for the Oct4-moderate cluster. Click here for file

Additional file 13 **Significant features**. This table lists significant features. Click here for file

Additional file 14 **Predicted target genes**. This table lists predicted target genes. Click here for file

Additional file 15 **Enriched GO terms for predicted targets**. This table lists enriched GO terms for predicted targets. Click here for file
